# Marine Terpenic Endoperoxides

**DOI:** 10.3390/md19120661

**Published:** 2021-11-25

**Authors:** Irene Torres-García, Josefa L. López-Martínez, Manuel Muñoz-Dorado, Ignacio Rodríguez-García, Miriam Álvarez-Corral

**Affiliations:** Organic Chemistry, University of Almería, ceiA3, E04120 Almería, Spain; itg491@ual.es (I.T.-G.); jlm216@ual.es (J.L.L.-M.); mdorado@ual.es (M.M.-D.); irodrigu@ual.es (I.R.-G.)

**Keywords:** marine terpenoid, endoperoxide, norseterterpene, norditerpene, sponge

## Abstract

Organic extracts of marine invertebrates, mainly sponges, from seas all over the world are well known for their high in vitro anticancer and antibiotic activities which make them promising sources of compounds with potential use as pharmaceutical leads. Most of the structures discovered so far have a peculiar structural feature in common: a 1,2-dioxane ring. This is a highly reactive heterocycle that can be considered as an endoperoxide function. Together with other structural features, this group could be responsible for the strong biological activities of the substances present in the extracts. Numerous research programs have focused on their structural elucidation and total synthesis since the seventies. As a consequence, the number of established chiral centres and the similarity between different naturally occurring substances is increasingly higher. Most of these compounds have a terpenoid nature, mainly diterpene and sesterterpene, with several peculiar structural features, such as the loss of one carbon atom. Although there are many reviews dealing with the occurrence of marine peroxides, their activities, or potential pharmaceutical uses, no one has focused on those having a terpene origin and the endoperoxide function. We present here a comprehensive review of these compounds paying special attention to their structural features and their biological activity.

## 1. Introduction

Marine water covers a great proportion of the earth’s surface and is proposed to contain a high percentage of the world’s plant and animal species; although, ninety one percent of marine species remain undiscovered [1]. Organic extracts of these marine species often exhibit promising specialized biological activities, prompting the search for new marine metabolites [2]. Between these compounds, a huge number of peroxides have been isolated, mainly from marine invertebrates, particularly in sponges and soft corals. 

Numerous peroxides have shown confirmed activity in vitro against tumour cells and they are considered an important source of leads for drug discovery [3]. In fact, there are actually three approved marine drugs derived from sponges and several more have entered clinical trial [4]. On the other hand, terpenoids have a great chemical structural diversity and are also important secondary metabolites of marine species presenting diverse bioactivities [5]. 

This review summarises marine endoperoxides of terpene origin and it is focused on their structure and the structural revision of newly-established stereochemistry, as well as in their biological activity. This article is structured into two main sections: the first one summarises endoperoxides with a norterpene skeleton (norsesteterpenes and norditerpenes, principally) and the second one summarises endoperoxides with a terpene skeleton (sesquiterpenes and diterpenes). The first section is also subdivided for a better understanding, based on the type of cyclic skeleton. A final overview of all reported biological activities for these systems is also included.

In all structural formulae, the stereocentres are depicted using the following convention: solid wedged bonds and hashed wedged bonds represent the known absolute configuration. In addition, *R* or *S* letters are written near the centre. When only the relative stereochemistry of the compound is known, it is represented with bold bonds and hashed bonds.

## 2. Marine Endoperoxide Norterpenes

### 2.1. Endoperoxide Norterpenes with an Acyclic Carbon Skeleton

**Figure 1:**
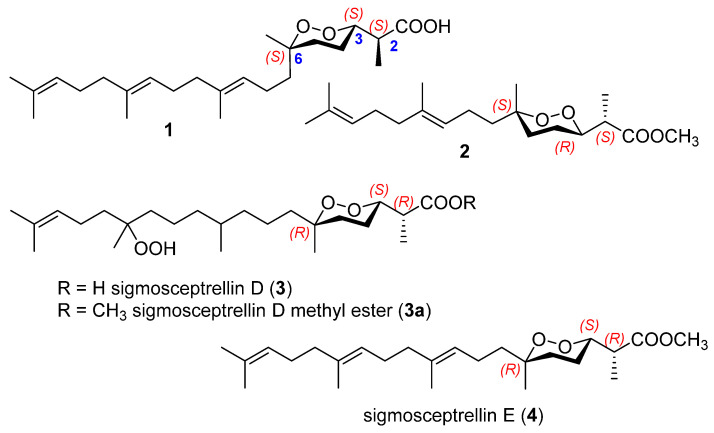
Endoperoxide norterpenes with an acyclic carbon skeleton (**1**–**4**).

The first two cyclic peroxides with an acyclic carbon skeleton (**1**, **2**), were described in 1985 by Capon and Macleod from an unidentified sponge collected from New South Wales [6]. Norsesteterpene **1** showed significant growth inhibitory activity against the gram-positive bacteria *Bacillus subtilis* and the yeast *Saccharomyces cerevisae*, while nordierpene **2** was inactive. Methylation of **1** yielded an inactive ester that was characterized as a peroxide with a *trans*-farnesyl unit; while compound **2** presented a *trans*-geranyl unit (Figure 1). Authors have determined the relative stereochemistry by NMR study and also on the basis of results of X-ray analysis of a crystalline derivative obtained from sigmoscetrellin A (see Section 2.3), which made it possible to establish the relative stereochemistry at C2, C3, and C6 [7]. Absolute stereochemistry of **1** and **2** was determined by application of Horeau’s procedure of asymmetric esterification [8].

Capon and MacLeod established an empirical rule based on ^1^H NMR chemical shifts for the C2 secondary methyl: in those cases where C2 and C3 are in an *erythro* configuration (*R*,*R* or *S*,*S*), the ^1^H NMR shift for the C2 methyl is upfield (δ 1.13–1.14 ppm), while in those examples where they are *threo* (*R*,*S* or *S*,*R*), this methyl resonates downfield (δ 1.24–1.26 ppm) [6]. On the other hand, in those examples were the C6 tertiary methyl is equatorial, the stereochemistries assigned to C3 and C6 are the same (*R*,*R* or *S*,*S*) while the opposite holds when the C6 methyl is axial. The former situation is consistent with the classical addition of oxygen across a *trans*, *trans* conjugated diene.

In 1999, two new examples of cyclic peroxides with an acyclic carbon skeleton were obtained from the crude EtOH extract of a *Sigmosceptrella* sp., which displayed growth inhibitory properties against some bacteria [9]. The norsesterterpene sigmosceptrellin D (**3**), characterized, as methyl ester (**3a**), was isolated from this extract. The low yield of sigmosceptrellin E (**4**) in the crude extract made impossible to establish whether it occurred naturally as either the carboxylic acid, the methyl ester, or both. These two norsesterterpene have a polyprenic chain like **1**, but present different stereochemistry.

The relative stereochemistry of the peroxide of **3a** was determined by application of empirical NMR rules, while the absolute stereochemistry was established using the Mosher procedure. The stereochemistry of the other two centres (C10 and C14) remain unassigned. It is worth mentioning that the hydroperoxide group of sigmosceptrellin D (**3**) is a rare functional group in marine natural products. Otherwise, the limited supply of **4** did not allow the determination of its absolute stereochemistry and 2*R*,3*S*,6*R* was tentatively proposed given the occurrence of related **3a**. 

**Figure 2:**
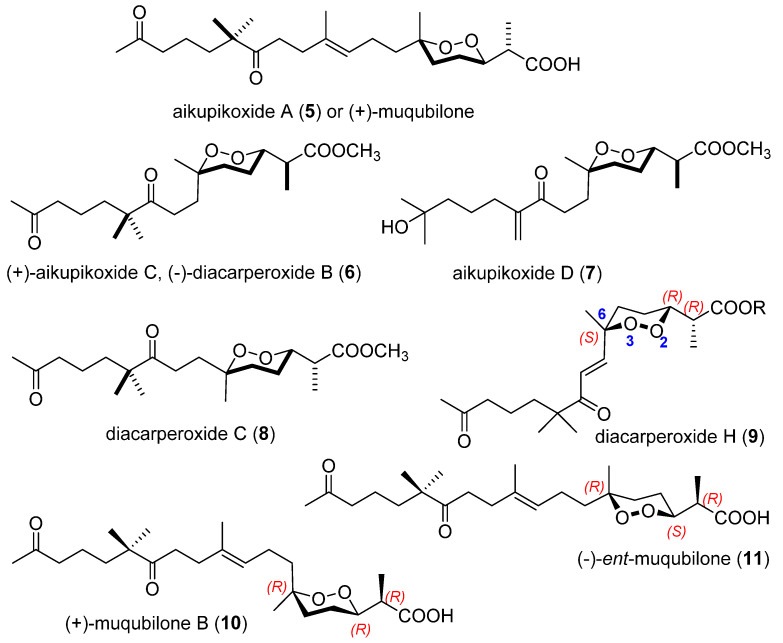
Endoperoxide norterpenes with an acyclic carbon skeleton (**5**–**11**).

In 2001, another norsesterterpene cyclic peroxide, aikupikoxide A (**5**), was isolated from the methanolic extract of the Red Sea sponge *Diacarnus erythraenus* as well as two norditerpenes aikupikoxides C and D (**6**, **7**) (Figure 2). These compounds showed cytotoxicity to mouse lymphoma (P-388), human lung carcinoma (A-549), and human colon carcinoma (HT-29) [10]. Aikupikoxide A (**5**) share the same endoperoxide ring as compound **2** (structure and stereochemistry) but it shows a unique structural feature by the presence of two ketone functional groups. In an independent study, the same compound was also isolated from the same sponge and in the same year, 2001, but it was named as muqubilone and showed in vitro antiviral activity against herpes simplex type [11]. The absolute configurations in **5** were not established in any of these two studies. Recently, in 2021, this compound (**5**) showed a moderate activity against the mouse lymphoma cell line L5178Y [12].

Closely related to **5**, norditerpenes aikupikoxides C and D also show oxygenation in the aliphatic side chain. The Formosan marine sponge, *Negombata corticata*, was also found to possess aikupikoxide C [13]. On the other hand, the enantiomer of aikupikoxide C (**6**), named as diacarperoxide B, was isolated from a sponge of the genus *Diacarnus* but from another species, *D. megaspinorhabdosa* [14]. Diacarperoxide B presented a negative [α]_D_ value while aikupikoxide C presented a positive one, but authors in both cases established just the relative stereochemistry. These two enantiomers are from two sponges of the same genus *Diacarnus* but different species. This is rare in marine natural products chemistry. Diacarperoxide C (**8**), a diastereoisomer of **6**, was found in *D. megaspinorhabdosa*.

Diacarperoxide H (**9**) was also isolated from *D. megaspinorhabdosa* collected in this case in the South China Sea. It has demonstrated in vitro antimalarial activity against *Plasmodium falciparum* (chloroquine resistant W2 clone) with IC_50_ values of 12.9 µM. Its structure, including conformations and absolute configurations, was determined by means of spectroscopic analyses, computational approaches and chemical degradation. This new endoperoxide presents one degree of unsaturation more than diacarperoxide B, and the absolute configuration 2*R*,3*R*,6*S* was determined by electronic circular dichroism [15].

Eight year later, two compounds structurally close to aikupikoxide A (**5**), named as (+)-muqubilone B (**10**) and (−)-*ent*-muqubilone (**11**), were isolated as carboxylic acids from *Diacarnus bismarckensis* [16]. These two compounds demonstrated activities in the range IC_50_ = 0.2–2 µg/mL against *Tripanosoma brunei*. Establishing the complete absolute configuration of **10** required NMR study and further transformation into Mosher’s derivatives of (+)-muqubilone B methyl ester. A 2*R*,3*R*,6*R* final absolute configuration was assigned, while compound **11** was assigned 2*R*,3*S*,6*R* using a process parallel to that just described for **10**. The observation of enantiomeric structures (**10** *versus* **5**) allowed a proposal for the absolute configuration of **5** (aikupikoxide A or (+)-muqubilone). These two enantiomers again came from the same genus *Diacarnus* but are different species. 

### 2.2. Endoperoxide Norterpenes with a Monocyclic Carbon Skeleton

The first norterpene-like endoperoxide isolated was muqubilin (**12**), described in 1979 by Kashman et al. [17] from the Red Sea sponge *Prianos* sp. Although in this work the stereochemistry of the molecule was not assigned, the monocyclic carbon skeleton of this norsesterterpene could be confirmed by ozonolysis, affording the same degradation product as the C_30_ isoprenoid mokupalide [18]. Until then, the cyclic peroxides found in sponges were largely derived from steroid structures or from the non-isoprenoid plakortin [19]. 

Several years later, in 1982, Solokoff et al. [20] isolated from Red Sea sponges a nor-sesterterpenoid peroxide that was named as prianicin A, although it was identical to muqubilin (**12**). The antibiotic properties of **12** against gram-positive and gram-negative bacteria and against fungi were measured, proving to be four to ten times more effective than tetracycline against beta hemolytic *Streptococcus* but non-effective against the gram-negative bacteria assayed.

**Figure 3:**
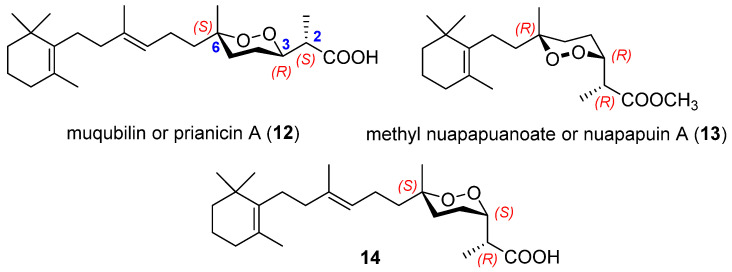
First isolated endopeoxide norterpenes with a monocyclic carbon skeleton.

Muqubilin (**12**) and methyl nuapapuanoate (**13**) (Figure 3) were isolated in 1984 from a drab sponge of Tonga coral reefs, an undescribed *Prianos* sp. [21]. The authors described the stereochemistry of muqubilin at C3 and C6, but not at C2, using NMR experiences and chemical correlation to known fragments. Besides that, a positive value for the optical rotation was described for **12**. It should be pointed out that the major contributions to the knowledge of the absolute configuration of this kind of compounds were made by Capon et al. [6]. An exhaustive study of NMR, chemical correlations, Horeau method, the use of α-phenylbutyric anhydride as agent for esterification, and X-ray analysis allow them to establish the configuration at C2, C3, and C6 for isoprene related endoperoxides. So, (+)-muqubilin (**12**) was definitely assigned as (2*S*,3*R*,6*S*). On the other hand, compound **14** (Figure 3) was isolated and described as a muqubilin diastereoisomer with the same absolute stereochemistry at C2, C3, and C6 than compound **1** (Figure 1). Both **12** and **14** were isolated from the same unidentified sponge Z4967 [6]. Ovenden et al. [9] also found peroxide **13** in *Sigmosceptrella* sp. from the Great Australian Bight, isolated as methyl ester. NMR data and optical rotation were identical to the product described as methyl nuapapuanoate but it was renamed as nuapapuin A (**13**). Using the established empirical rules and an advanced Mosher procedure, configuration of nuapapuin A (**13**) was confirmed as (2*R*,3*R*,6*R*). The authors proposed a biosynthetic pathway to explain the formation of the 3,6-*trans* and 3,6-*cis* norterpene peroxides. Nuapapuin A exhibits cytotoxicity towards cancer cell lines SW480, NCI-H460, PC-9, and HepG2, with IC_50_ 3.7, 7.8, 6.2, and 8.0 µM values respectively [22]. 

Muqubilin (**12**) was also examined for in vitro activity against the Da and W2 clones of *Plasmodium falcipanum*, proving to be a good prototype for the development of new antimalarial agents. The authors suggest that the activities of this compound and the well-known artemisinin may be attributed to their peroxide functional groups [23,24]. On the other hand, muqubilin showed no insecticidal activity against larvae of corn rootworm and tobacco budworn [25]. More recently, muqubilin was also found in the Red Sea sponge *Diacarnus erythreanus* [11]. It showed in vitro antiviral activity against herpes simplex type 1 and also potent in vitro activity against *Toxoplasma gondii* without significant toxicity. Muqubilin has also been examined in a program to identify marine natural products as prototype agrochemical agents. It showed moderate activity against *Nicotiana tabacum* [26]. 

**Figure 4:**
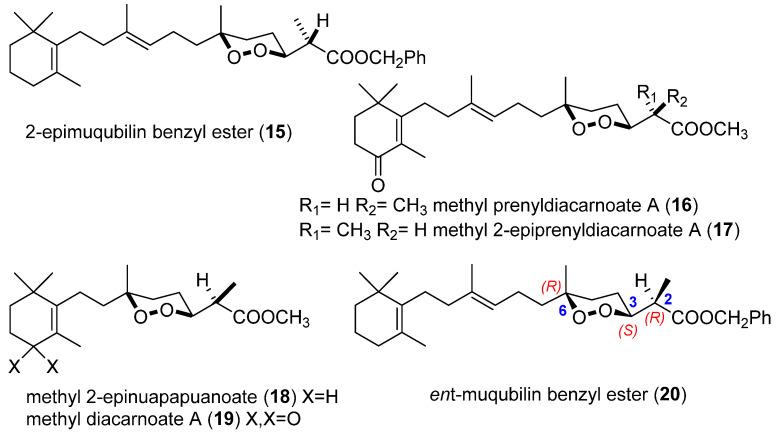
Terpenoids isolated from the sponge *Diacarnus levii*.

From the sponge *Diacarnus levii*, collected along the outer reef in front of Noumea, New Caledonia, five endoperoxide terpenoid acids were esterified, and the esters (**15**–**20**) were purified (Figure 4) [27]. The relative configurations at C2, C3, and C6 were assigned. It is noteworthy to mention that compounds **15** and **18** were named as 2-epimuqubilin and 2-epinuapapuanoate, respectively, although it would be more accurate to use alternative terms, because they are diastereomers but not epimers at C3. The marked cytotoxicity of norsesterterpenoids **15**, **16**, and **17** in comparison with the inactivity of the respective norditerpenoids **18** and **19** towards KB tumoral cell lines suggests that the length of the lipophilic chain could be important. A possible explanation could be the lipophilicity effect that should allow the norsesterterpenoids to cross cell membranes. However, judging from the similar bioactivities of **15**–**17**, the α,β-unsaturated ketone seems not to be essential [27]. One year later, the same authors [28] isolated, after esterification, *ent*-muqubilin benzyl ester (**20**). Its absolute configuration was determined by the Mosher’s NMR methodology. They also found that compounds **15**, **18**, **19**, and **20** were active in vitro against the malaria parasite *Plasmodium falciparum*, especially **18**, active against a chloroquine-resistant strain with a good security index [24,28]. Compound **18** and its enantiomer were also purified from another sponge, *Diacarnus erythreanus*, collected from the Jordan coast [12]. Compound **18** exhibited pronounced cytotoxic potential against the murine lymphoma (L5178Y) cell line with IC_50_ values of 6.4 μM. Molecular docking studies of these compounds suggest potential EGFR (epidermal growth factor receptor) inhibition. The analysis of two *Thorectidae* sponges, *Hyrtios* sp. and *Petrosaspongia* sp., collected at the Fiji Islands [29], led also to the isolation of methyl diacarnoate A (**19**) [27]. 

**Figure 5:**
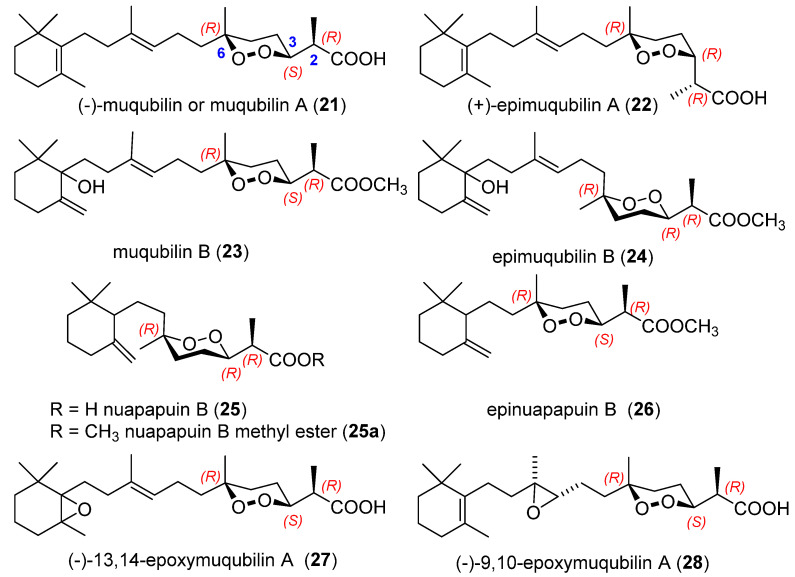
Norterpenoids isolated from sponges of genus *Diacarnus*.

Crews and coworkers [30] studied the composition of the extracts of the marine sponge *Diacarnus* cf. *spinopolum* (revised from *Prianos* sp.) and described two isomers of muqubilin (**12**). One of them is its enantiomer, with a 2*R*,3*S*,6*R* configuration and named as (−)-muqubilin or muqubilin A (**21**). The other is a diastereomer named as epimuqubilin A (**22**) with a 2*R*,3*R*,6*R* configuration (Figure 5). Besides, they characterized two norsesterterpene isomers hydroxylated in C13, muqubilin B (**23**), and epimuqubilin B (**24**), the norditerpenoids nuapapuin B (**25**) and its methyl ester (**25a**) and epinuapapuin B (**26**) (Figure 5). Nuapapuin B was chemically converted to methyl nuapuanoate [21] (**13**) and, for both compounds, (2*R*,3*R*,6*R*) configuration was established. Muqubilin A proved to be a cytotoxic compound, although lacking selectivity between normal and cancer cells assays of in vitro growth inhibition. It induced ROS (reactive oxygen species) production in cancer cells, while no apoptosis was observed in the same cells treated by this compound [31]. In 2019, D’Aniello et al. [32], in a project aiming to discover new pharmacological tools to treat neurological diseases and cancers, identified (−)-muqubilin (**21**) as the first example of an allosteric modulator of RARα, using a combination of computational and experimental approaches.

Epimuqubilin A (**22**) and epinuapapuin B (**26**) methyl ester were also obtained from the sponge *Diacarnus bismarckensis* [16]. These peroxiterpenes presented activity against *Trypanosoma brucei*, the causative agent of African sleeping sickness (IC_50_ = 0.2–2 µg/mL), being templates for the development of therapeutic leads. In 2010, Ghang’s group [33] determined that (+)-epimuqubilin A (**22**), isolated from the marine sponge *Latrunculia* sp., possesses potent NO inhibitory activity against lipopolysaccharide (LPS)-induced nitric oxide release with an IC_50_ value of 7.4 µM, a level three times higher than the positive control, L-N^G^-monomethyl arginine citrate. The study of structure-activity with several norsesterterpene peroxides indicated a higher activity of the free acids *versus* the esters, as well as a monocyclic carbon skeleton being essential for the activity. However, compounds with a bicyclic structure had a reduced activity. Lately, in 2011, the same group [34] found that (+)-epimuqubilin A (**22**) reduced iNOS and COX-2 expression through inhibition of the NF-κB pathway, specifically by blocking the activation of IKKβ. This is a unique molecular mechanism to warrant further exploration of this peroxide as potential anti-inflammatory or cancer chemopreventive agent.

The epoxides, (−)-13,14-epoxymuqubilin A (**27**) and (−)-9,10-epoxymuqubilin A (**28**) (Figure 5) were characterized from the Red Sea sponge, *Diacarnus erythraeanus*, together with the known marine metabolites nuapapuin A methyl ester (**13**), methyl 2-epinuapapuanoate (**18**), and muqubilin A (**21**) [31]. Based on biogenetic considerations, the absolute configuration at C2, C3, and C6 in both compounds were assumed to be the same as that in muqubilin A (**21**), but the configuration of oxirane ring of **27** remained undetermined. 

**Figure 6:**
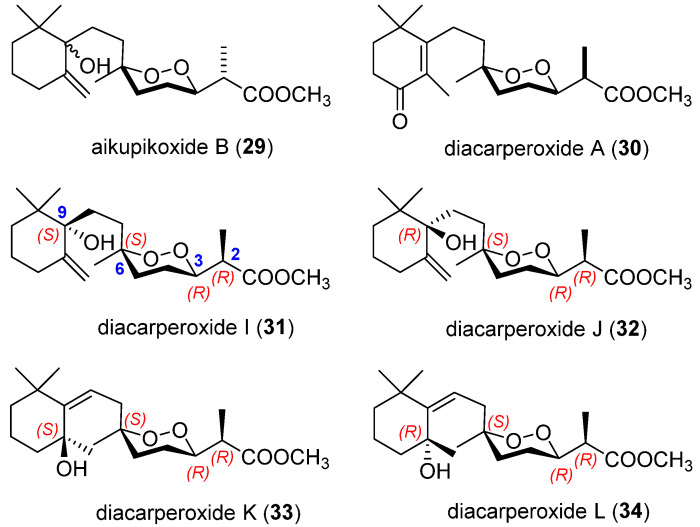
Norditerpenes isolated from the genus *Diacarnus*.

In addition to all the compounds shown in Figure 5, the genus *Diacarnus* has proven to be a good source of norditerpenes (Figure 6). From the lipophilic extract of the Red Sea sponge *Diacarnus erythraenus* [10], a norditerpene cyclic peroxide, named aikupikoxide B (**29**), was isolated. The assignment or the relative configuration at C2, C4, and C6 was based in the empirical rules introduced by Capon et al. [6]. 

The hexane extract of the sponge *Diacarnus megaspinorhabdosa* provided a series of norterpene derivatives including diacarperoxide A (**30**) [14] and the known products nuapapuin A methyl ester (**13)**, methyl 2-epinuapapuanoate (**18**), methyl diacarnoate A (**19**), epimuqubilin B (**24**), and compound **14**. The relative configuration of chiral centres at C2, C3, and C6 was assigned by established empirical rules. Diacarperoxides I–L (**31**–**34**) have also been described from another *Diacarnus* sponge, *D. megaspinorhabdosa*, collected from the South China Sea in 2014 [15]. The structures of these norditerpene compounds were determined by the use of spectroscopic analyses and computational approaches, NMR, ECD (electronic circular dichroism), and conformational analysis calculations. Diacarperoxides I (**31**) and J (**32**) showed in vitro antimalarial activity.

**Figure 7:**
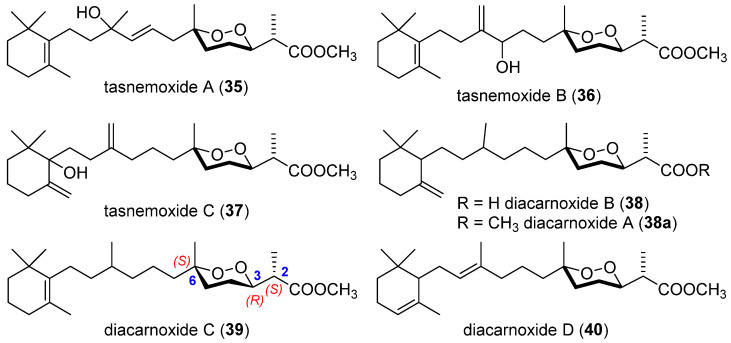
Tasnemoxides and diacarnoxides form *Diacarnus* genus.

Following the study of the genus *Diacarnus*, another two groups of norsesterterpenes have been described from these sponges: tasnemoxides and diacarnoxides (Figure 7). After exclusion chromatography, flash ODS column and normal- and reversed-phase HPLC purification the norsestertepenes tasnemoxides A–C (**35**–**37**) were afforded [35] from the Red Sea sponge *Diacarnus erythraenus*. The assignment of relative stereochemistry and connectivities were supported by HMBC experiments. 

Dai in 2007 [36] studied the lipid extract of the Papua New Guinea marine sponge *Diacarnus levii*. Four new norsesterterpene peroxides were described, diacarnoxides A–D (**38**–**40**) and the relative configuration of their chiral centres was assigned. Diacarnoxide B (**38**) and its methyl ester **38a** exhibit a significantly enhanced ability to suppress the growth of tumour cells under hypoxic conditions. In 2017, Schneider and Seifert [37] described the first total asymmetric synthesis of diacarnoxide C (**39**). They achieved the production of an inseparable mixture of diacarnoxide C, (2*S*,3*R*,6*S*,10*RS*) together with its 6*R*-epimer. In this way, the absolute stereochemistry of the natural product could be established.

**Figure 8:**
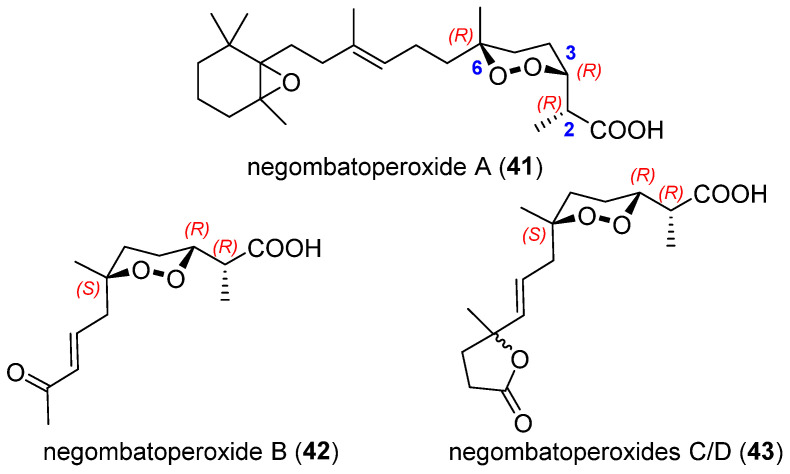
Cyclic peroxides isolated from *Negombata corticata*.

The study of the marine sponge, *Negombata corticata* allowed the isolation of three norterpene-related cyclic peroxides: negombatoperoxide A (**41**) and B (**42**) and an inseparable epimeric mixture of negombatoperoxides C and D (**43**) (Figure 8). Negombatoperoxide B has an odd 13-carbon skeleton and it has been included in this subheading because of its analogies with the two others negombatoperoxides. On the basis of biogenetic considerations, the absolute configuration of these compounds was tentatively proposed to be the same as in (+)-nuapapuin B (**25**), which was also isolated from this sponge [13]. Cytotoxicity of these compounds against human breast carcinoma, human liver carcinoma, and human lung carcinoma cell lines was evaluated. Compounds **41**–**43** did not show activity but **25** and its methyl ester (**25a**) were active against these cell lines.

**Figure 9:**
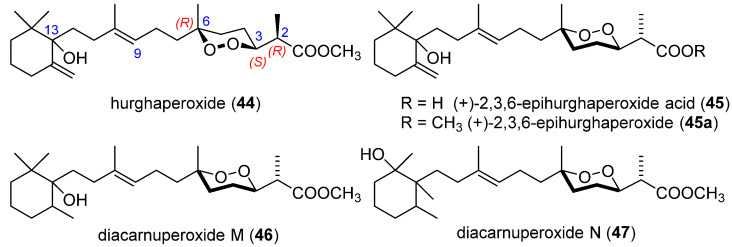
Norsesterterpene (**44**–**47**) cyclic peroxides.

In 1996, Guo et al. [38] described a new norsesterterpene cyclic peroxide, named hurghaperoxide (**44**) from an undescribed Red Sea sponge, hydroxylated in the cyclohexane ring. By comparison with other molecules and also by chemical correlation, the absolute configuration of the first six carbons could be established, although not at C13 (Figure 9). This compound is a diastereomer of muqubilin B (**23**). In 2016, four similar norterpene cyclic peroxides (**45**–**47**) (Figure 9) were isolated from the Xisha Islands sponge *Diacarnus megaspinorhabdosa* [39]. Their structures were elucidated on the basis of spectroscopic analyses and comparison with related model compounds. (+)-2,3,6-epihurghaperoxide acid (**45**) is the enantiomer of hurghaperoxide (**44**), while diacarnuperoxide M (**46**) differs in the exocyclic double bond, and diacarnuperoxide N (**47**) in the position of the hydroxy group. Stereochemistry at C2, C3, and C6 in the last three compounds is the same as in diacarnoxides (**38**–**40**) (Figure 7). Muqubilin A (**21**), nuapapuin A (**13**), and diacarperoxide A (**30**) were also isolated from this sponge. All these compounds (**13**, **21**, **30**, **44**, **45**, **45a**, **46**, and **47**) were evaluated for the inhibitory activity against the malaria parasite *Plasmodium falciparum*, and all of them showed significant antimalarial activity with IC_50_ values in the range of 1.6–8.6 mM. 

### 2.3. Endoperoxide Norterpenes with a Bicyclic Carbon Skeleton

**Figure 10:**
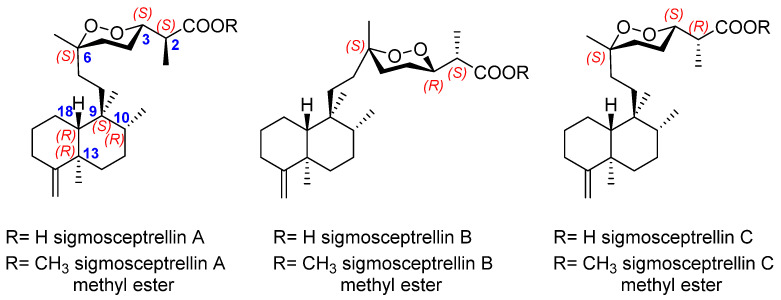
Sigmosceptrellins A–C: first described structures and absolute stereochemistry.

The norsesterterpene peroxides sigmosceptrellin A and B were reported for the first time in 1979 by Albericci et al. [7]. A third norsesterterpene peroxide sigmosceptrellin C was isolated years later by the same authors [40] from the ichthyotoxic fraction of the sponge *Sigmosceptrella laevis* (Lindgren), collected in Papua New Guinea. These three sigmosceptrellins were isolated as synthetic methyl ester derivatives, although the natural esters of A and B compounds also appeared as minor constituents in the sponge extracts (Figure 10). 

Sigmosceptrellin A was the first example of a norsesterterpene with a non-isoprenic carbon skeleton with a decalin fragment identical to that of other sponge metabolites (e.g., avarol [41,42]). The structure and relative configuration of sigmosceptrellin A were deduced by X-ray diffraction analysis of a crystalline derivative [7], while sigmosceptrellin B and sigmosceptrellin C were first deduced by chemical intercorrelations [40]. In 1982, Piccini-Leopardi et al. [43] confirmed the structure of sigmosceptrellin B by X-ray method. These three sigmosceptrellins were described as stereoisomers, suggesting that the stereochemical changes are confined to the 1,2-dioxane ring. While isomer A has an *erythro* configuration (C2 and C3), isomers B and C have a *threo* configuration at those carbon atoms. Chemical interrelation of isomers B and C with A secured their relative configurations [6]. 

**Figure 11:**
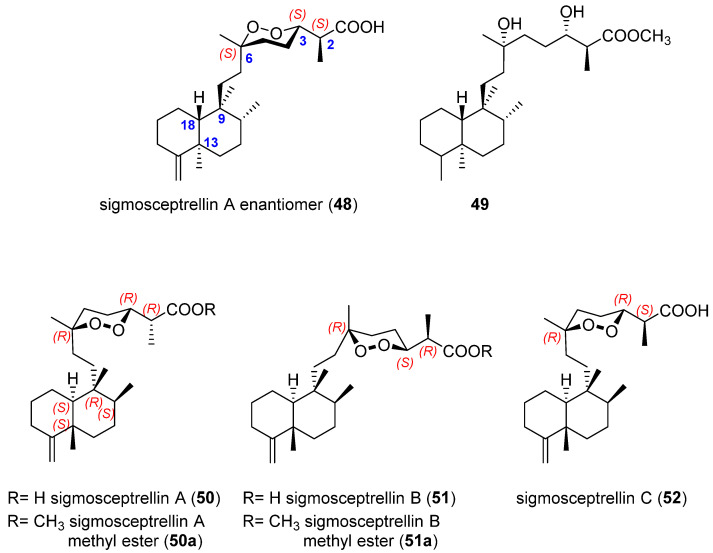
Sigmosceptrellin A enantiomer (**48**) and its hydrogenated derivative (**49**) and revised absolute stereochemistry of sigmosceptrellin A–C (**50**–**52**) [6].

However, in 1985, Capon and MacLeod [6] published a revision of the assigned absolute stereochemistry of these sigmosceptrellins. A sponge, Z4969, collected at the same locality as that described by Abericci et al. contained the norsesterterpene peroxide **48**, which was isolated as its methyl ester (Figure 11). Its spectroscopic characteristics were identical to those of sigmosceptrellin A, but with an opposite optical rotation. Therefore, it was established as its enantiomer. In this case, the authors decided to study the absolute stereochemistry by application of Horeau’s procedure [8] of asymmetric esterification on its hydrogenation product (**49**) and it returned a C3 stereochemistry (*S*) opposite to that expected (*R*) by correlation with its enantiomer (Figure 11). A similar procedure was carried out with sigmosceptrellin A and a 3*R* stereochemistry was established. A possible explication is that the stereochemical assignment based on CD observation was incorrect, and these authors made new reassignment of absolute stereochemistry for sigmosceptrellin A (**50**) (2*R*,3*R*,6*R*), sigmosceptrellin B (**51**) (2*R*,3*S*,6*R*), and sigmosceptrellin C (**52**) (2*S*,3*R*,6*R*) (Figure 11).

In 1996, El Sayed et al. [23] also isolated sigmosceptrellin A (**50**) from the lipophilic extract of a sponge of the Red Sea originally identified as a *Prianos* sp., but revised to *Diacarnus* cf. *spinopoculum* [44]. In vitro testing of sigmosceptrellin A showed that it can selectively inhibit *P. faliciparum*, a malarial parasite, with an IC_50_ = 470 ng/mL. This sigmosceptrellin was also isolated from a *Latrunculia* sp. C006879 and exhibited significant inhibitory activities against yeast GSK-3β plates [33]. 

Sigmosceptrellin B (**51**) and its ester (**51a**) were also identified in the Red Sea marine sponge *Diacarnus erythraeanus*, collected in the Gulf of Aqaba, Jordan [12]. These compounds exhibited pronounced cytotoxic potential against murine lymphoma (L5178Y) cell line with IC_50_ values of 1.2 (**51**) and 3.4 (**51a**) µM. It was found that **51** exhibits more potent cytotoxic activity than **51a**, which might be attributed to the latter’s hydrophobic properties and steric hindrance posed by the inserted methyl group. Molecular docking studies of these compounds have also been conducted, suggesting possible epidermal growth factor receptor (EGFR) inhibition. In addition, **51** showed in vitro antimalarial activity against *P. falciparum* (D6 and W2 clones) and displayed potent in vitro activity against *Toxoplasma gondii* at nontoxic concentrations [11].

From the sponge *Diacarnus bismarckensis*, sigmosceptrellin A (**50**), sigmosceptrellin A methyl ester (**50a**), and sigmosceptrellin B (**51**) were isolated. These peroxiterpenes demonstrated activity against *Trypanosoma brucei*, a protozoan parasite causative of human African trypanosomiasis (HAT), also known as African sleeping sickness [16].

**Figure 12:**
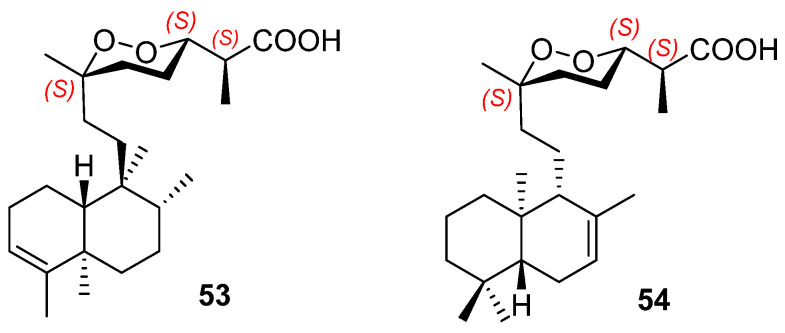
Sigmosceptrellins from Mycale ancorina.

**Scheme 1:**
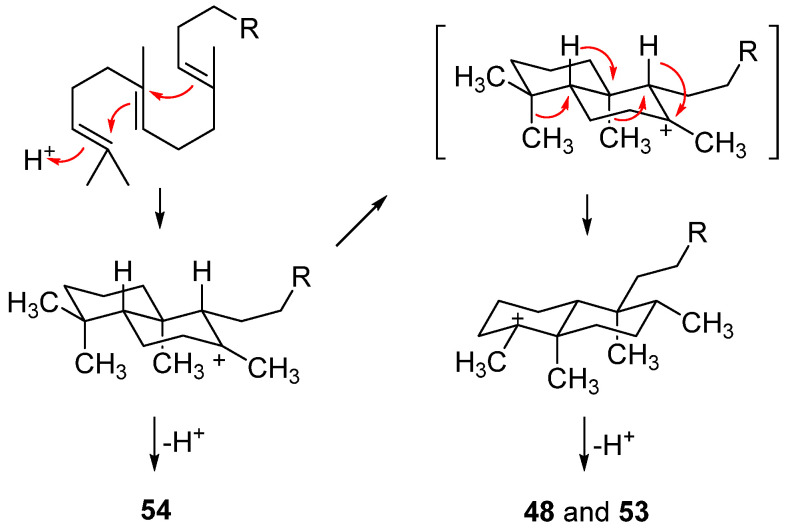
Proposed biosynthetic sequence leading to the bicyclic ring systems.

In 1987, two new sigmosceptrellins, **53** and **54**, were isolated as their methyl esters from *Mycale ancorina* Whitelegge (Figure 12) [45]. The major isomer **53** was identified as the endocyclic double bond isomer of enantio-sigmosceptrellin A (**48**). The minor component **54** has the same cyclic peroxide moiety as **53**, but the bicyclic portion is a *trans*-labdane type bicyclic unit rather than a clerodane-type. Its absolute stereochemistry would support a 2*S*,3*S*,6*S*,9*R*,13*R*,18*S* and is consistent with a common biosynthetic sequence leading to the bicyclic ring systems in the marine natural products enantio-sigmosceptrellin A (**48**), **53** and **54** (Scheme 1) [45]. 

**Figure 13:**
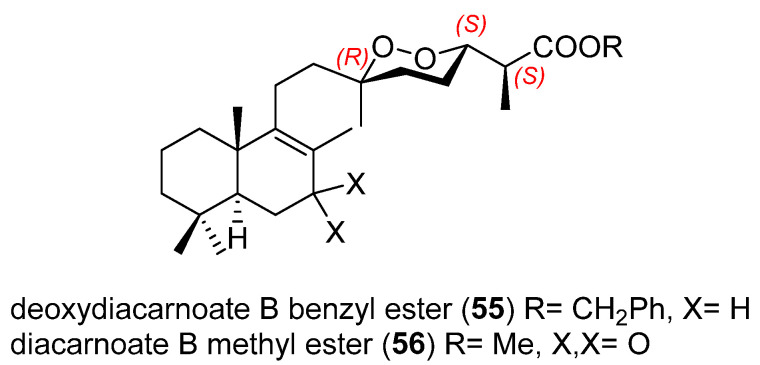
Epidioxy-norsesterterpenes from *Diacarnus levii*.

Deoxydiacarnoate B benzyl ester (**55**) and diacarnoate B methyl ester (**56**) (Figure 13), two cyclic norditerpene peroxides, were isolated after esterification of natural free carboxylic acids from the hadromerid sponge *Diacarnus levii*, collected off New Caledonia waters [28]. Compound **55** showed substantial in vitro activity against the chloroquine-resistant *Plasmodium falciparum* strain, thus having potential as a new antimalarial lead. The relative configuration of the peroxide moiety in **55** and **56** was determined from their NMR spectra. In particular, the chemical shifts support an *erythro* configuration at C2 and C3. The absolute configuration of the peroxide/α-methylacetate moiety (2*S*,3S,6R) was deduced using its phenylglycine methyl ester derivates and comparing ^1^H NMR data. The absolute configuration at the carbocyclic moiety of **55** and **56** is identical, as established by chemical interconversion. According to empirical CD rules and the synthesis of a model, the authors suggested the labdane configuration for **55** and **56**.

**Figure 14:**
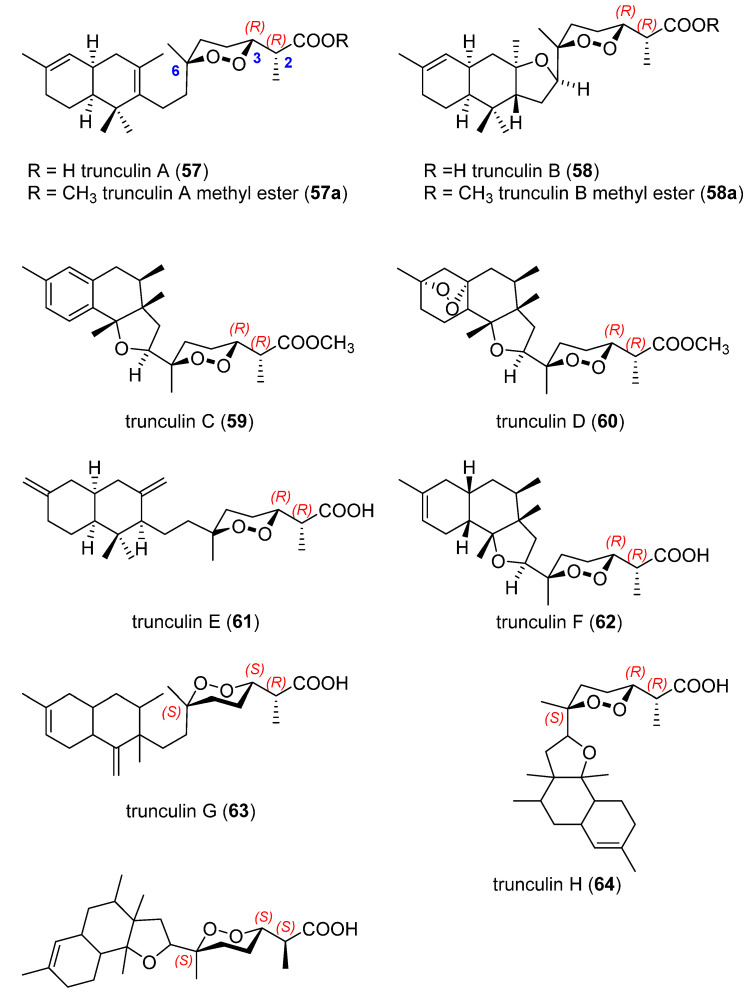
Chemical structures of trunculins A–I.

In 1987, Capon et al. [46] described the structural elucidation of two new norsesterterpene cyclic peroxides possessing a new carbon skeleton, trunculin A (**57**) and trunculin B (**58**), that together with their methyl esters (**57a**–**58a**) were isolated from the Australian marine sponge, *Latrunculia brevis* (Figure 14). Compounds **57** and **58** showed significant growth-inhibitory activity against the gram-positive bacteria *Bacillus subtilis* and “yeast” *Saccharomyces cerevisae*, while their corresponding methyl esters were inactive. 

Four years later, three more trunculins were found in a specimen of the marine sponge *Latrunculia* sp. from Jervis Bay, Australia: trunculin C methyl ester (**59**), trunculin D methyl ester (**60**), and trunculin E (**61**) (Figure 14) [47]. Antimicrobial assays performed on this series of peroxides showed that the antimicrobial activity of the crude extracts of *Latrunculia* sp. is associated with the free acids but not with the corresponding methyl ester. Therefore, only trunculin E (**61**) inhibits the growth of *Staphylococcus aureus*, *Bacillus subtilis*, and *Candida albicans* when tested at 100 µg/disk in the standard disk assay. 

Trunculin F (**62**), an additional very unstable norsesterterpene acid, was identified as its methyl ester (Figure 14) from Latrunculia conulosa, an Australian specimen [48]. A Latrunculia sp. collected off Philip Bay, Victoria, contained three new trunculins; trunculin G (**63**), H (**64**), and I (**65**) were isolated as their methyl esters for better study (Figure 14) [49]. The crude ethanolic extract of this sponge displayed growth inhibitory properties against Serratia marcescens and Staphylococcus aureus. 

In order to establish the relative configuration of these compounds, the empirical rules established by Capon and MacLeod [6] were applied, focusing on the ^1^H NMR shift of the C2 secondary methyl (*erythro* ~ δ 1.14 and *threo* ~ δ 1.25). All the trunculins, except trunculin G, have an *erythro* configuration between C2 and C3. The complete relative stereochemistry of trunculin A, B, and C (**57**–**59**) was determined by single-crystal X-ray crystallographic experiments. In addition, the 3*R* absolute stereochemistry of these trunculins was determined by asymmetric esterification (Horeau analysis [8]) of the saturated diol esters derivative.

Almost all the chiral centres in trunculin D (**60**) could be defined as a result of its facile conversion into trunculin C (**59**) upon treatment with *p*-toluenesulfonic acid in benzene. The configuration of the peroxide bridge was defined by a single nuclear Overhauser enhancement experiment in which irradiation of the C18 methyl signal caused strong enhancements of the C16 methylene signals. Detailed analysis of the ^1^H and ^13^C NMR spectra of trunculin E and F (**61**–**62**), as well as the comparison of these data with those due to trunculins A, B, and C, allowed the assignment of the relative configuration of these compounds. In addition, Horeau analysis [8] on the respective diols established an *R* absolute stereochemistry at C3. For trunculins G, H, and I (**63**–**65**), the relative and absolute configuration of the cyclic peroxide moiety could be established by spectroscopic methodology and by the use of both the Horeau [8] and Mosher procedures [50]. However, the stereochemistry at the chiral centres of the decalin system could not be unambiguously defined. 

**Scheme 2:**
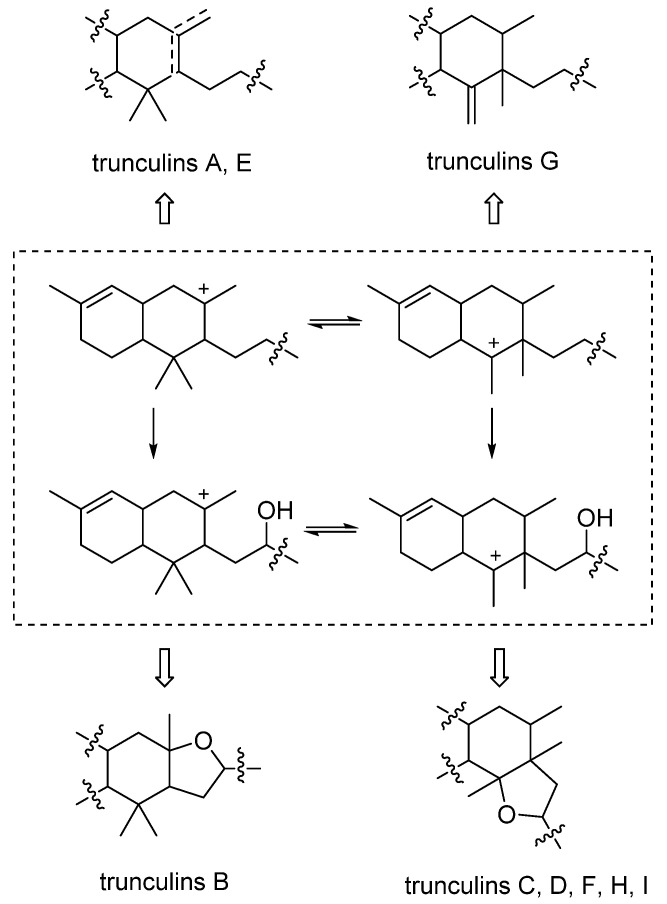
Plausible biosynthetic relationships between known trunculins.

A plausible biosynthetic relationship could be proposed by comparison of the nine trunculins (Scheme 2). The significance of the tertiary carbocations capable of facilitating both the 1,2-methyl migration or the cyclization to yield tetrahydrofurans [49] is noteworthy. 

**Figure 15:**
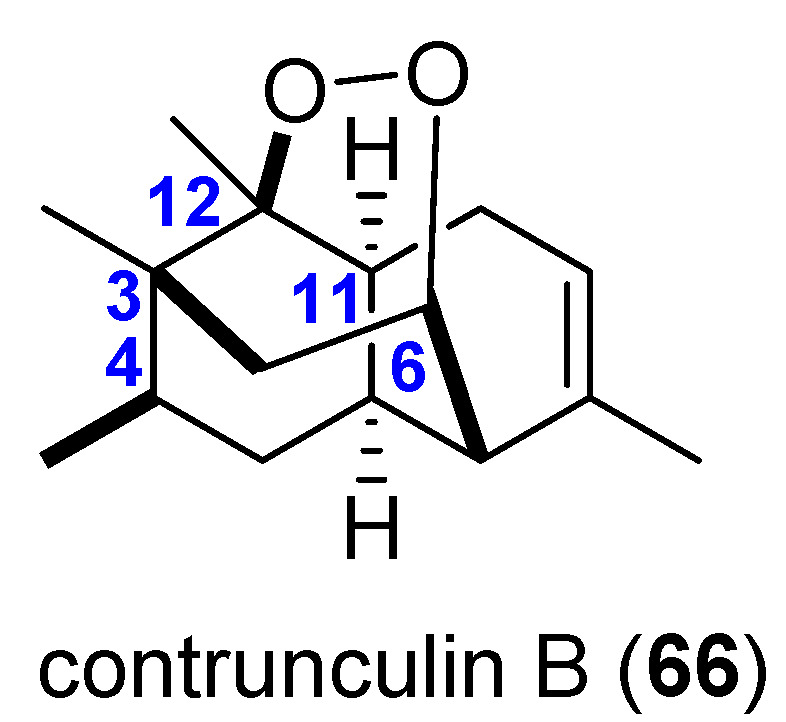
Structure of tricyclic norterpene contrunculin B (**66**).

Exceptionally, a tricyclic endoperoxide contrunculin B (**66**) (Figure 15) was isolated from the Australian marine sponge *Latrunculia conulosa* [48]. The structure was established by spectroscopic data. The relative stereochemistry was determined by NOE difference experiments. Given its structural similarity to trunculin F (**62**), and the instability of the polar material in the crude extract, it is not clear whether it is a natural product or an artifact produced during the extraction or the isolation process. However, no traces of this compound were detected in the decomposition products of an authentic sample of trunculin F methyl ester (**62a**) when it underwent decomposition.

**Figure 16:**
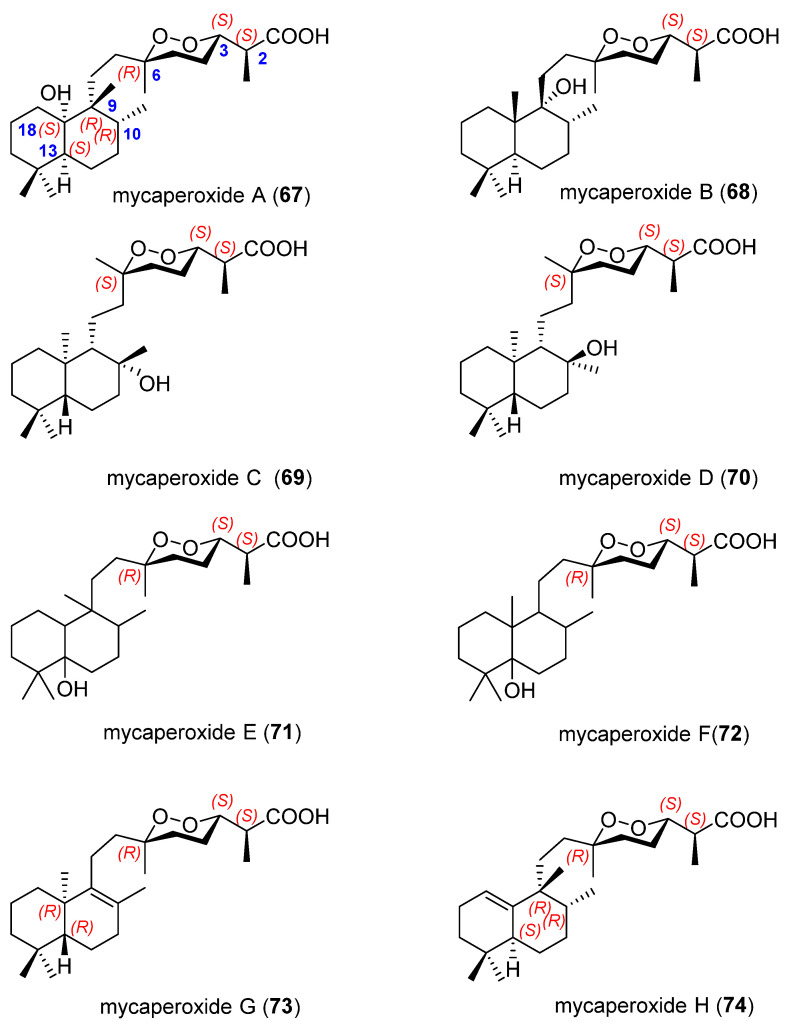
Norterpene cyclic peroxides with a bicyclic carbon skeleton from *Mycale* sp.

The two norsesterterpene, mycaperoxide A (**67**) and mycaperoxide B (**68**) (Figure 16), were first isolated from *Mycale* sp. sponge in Thai bay by Tanaka et al. in 1993 [51]. Both compounds were obtained from the methanolic extract, after different chromatographic separations. An X-ray analysis was made to determine the structure of **67**. Chemical correlations between both mycaperoxides **67** and **68** suggested the same configuration at C2, C3, C6, C10, and C13. Their absolute stereochemistry was assigned using the Kusumi and Kakisawa modification of Mosher’s method [52]. These compounds inhibited the growth of the gram-positive bacteria *Bacillus subtilis* and *Staphylococcus aureus*, and also showed cytotoxicity against the cell lines P-388, A-549, and HT-29. Besides, antiviral activity against herpes simplex virus type-1 have been described for mycaperoxides A and B.

Four years later, Capon et al. isolated two new norsesterterpenes of this family of compounds, mycaperoxide C (**69**) and mycaperoxide D (**70**) as methyl esters, from an Australian *Mycale* sp. sponge (Figure 16) [53]. The structure of both isomers was elucidated by NMR spectroscopy comparison with the known micaperoxides A (**67**) and B (**68**), and other marine products previously described. On the basis of the optical properties of **69**, using the molar rotation contributions for the two chiral subunits of the structure are additive [45], the absolute stereochemistry (2*S*,3*S*,6*S*,9*S*,10*R*,13*R*,18*S*) was assigned. This stereochemistry is consistent with a common biosynthetic sequence with **67** and **68**. It was not possible to propose an absolute stereochemistry for **70**, but a tentative assignment was made on the basis of a common biosynthetic origin.

Mycaperoxide E (**71**) and mycaperoxide F (**72**) (Figure 16) were first isolated as methyl esters from the active ethanolic extract of an Australian *Mycale* (Carmia) cf. *spongiosa* in 1991 [54], but they were named and the stereochemistry of the peroxide ring in **72** was revised in 1998 [55]. However, none of these two works described the absolute or even the relative configuration of the decalin ring system. Mycaperoxide G (**73**) was also isolated as its methyl ester from another *Mycale* sp. (F77046) from the Great Australian Bight [55]. A comparison of NMR data between the norsesterterpenes mycaperoxides E (**71**) and F (**72**) methyl esters and mycaperoxide G (**73**) methyl ester revealed a common cyclic peroxide moiety, but different bicyclic subunits. The stereochemistry of the bicyclic system of **73** was achieved by NMR comparation with known marine natural products also isolated from Australian sponges [56], and using molar rotation described in literature [57]. The absolute stereochemistry at C3 was experimentally established by application of the advanced Mosher procedure [50]. 

In 2003, a bioassay-guided isolation afforded a new norsesterterpene peroxide, mycaperoxide H (**74**) (Figure 16), from a Thai marine sponge *Mycale* sp. [58]. This natural product was cytotoxic against HeLa cells with an IC_50_ value of 0.8 µg/mL. Furthermore, through theoretical studies based on the protein–protein interaction site, it was predicted that mycaperoxide H can induce cytotoxicity in corneal endothelial cells through the blocking of the antagonist site of the sFRP1 protein, thus helping to prevent glaucoma [59]. Its structure was determined with 1D and 2D NMR experiments and the absolute configuration of the cyclic peroxide moiety (2*S*,3*S*,6*R*) was assigned by Mosher analysis [50]. In addition, the chemical conversion of **68** into **74** confirmed its stereochemistry.

**Figure 17:**
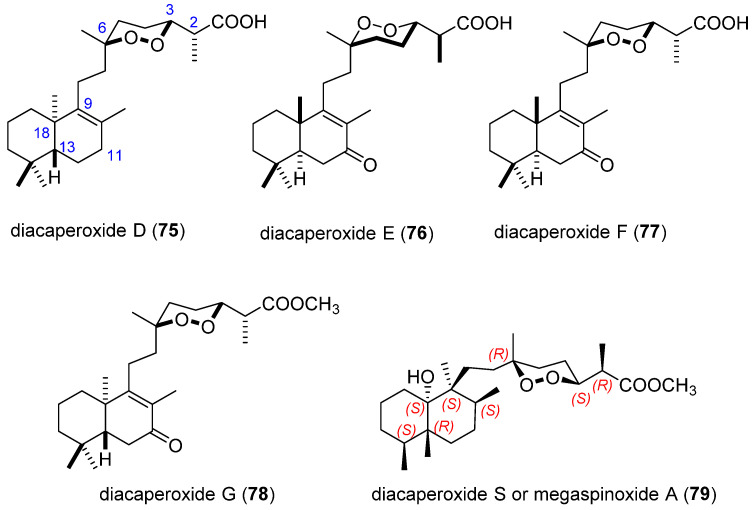
Norterpene cyclic peroxides with a bicyclic carbon skeleton from *Diacarnus megaspinorhabdosa*.

The methanolic extract of the Indonesian sponge *Diacarnus megaspinorhabdosa* afforded four norsesterterpene bicyclic peroxides, diacaperoxides D, E, F, and G (**75**–**78**) (Figure 17), besides other previously reported peroxides [14]. Strong or moderate cytotoxic activity has been found for these compounds, in particular, against L5178Y mouse lymphoma, HeLa human cervix carcinoma and PC12 rat brain tumour cells (except diacaperoxide E (**76**) in the last two cases). Authors pointed out that the insertion of a prenyl unit in the central portion of the molecule made these norsesterterpenes cyclic peroxides more active than their norditerpene congeners. 

The relative stereochemistry of the decalin system of diacaperoxide D (**75**) could not be elucidated by NMR, due to overlapping resonances in its NOESY spectra. Using the empirical rules described by Capon and Macleod [6] and the molar-rotation additivity rule [60], it was established to be 2*R*,3*R*,6*R*,13*R*,18*R*, confirming an *ent*-labdane moiety. The ^13^C NMR data of diacaperoxide E (**76**) was comparable to those of **75** except for a signal at δ_C_ 200.7 ppm corresponding to the additional carbonyl carbon. Besides, the NMR data correlate with those of diacarnoate B methyl ester (**56**) (Figure 13). The only difference was the equatorial orientation of CH_3_-20 in **76**, which was axially oriented in **56**. The relative stereochemistry of **76** was assigned following the strategy described above as (2*S*,3*S*,6*S*,13*S*,18*S*), being the decalin moiety characterized in this case as a labdane unit.

Inspection of the NMR spectral data and the optical properties of diacaperoxide F (**77**) led to the conclusion that **77** is a diastereoisomer of **76**, with the same carbon skeleton but different chiral centres in the cyclic peroxide moiety (2*R*,3*R*,6*R*). On the other hand, diacaperoxide G (**78**) is a methyl ester, whose relative configuration was established as (2*R*,3*R*,6*R*,13*R*,18*R*). 

Diacaperoxide S (**79**) (Figure 17) was isolated in 2012 [61], after reinvestigating the methanolic extract of the Indonesian sponge *Diacarnus megaspinorhabdosa*. Like their congeners, **79** showed strong activity against the same cancer cell lines, as well as strong activity against *Bacillus cereus*, *Staphylococcus aureus*, and *Candida albicans*. This new norsesterterpene was compared with the well-known sigmosceptrellin B (**51**) (Figure 11), and it proved to have the same skeleton except for the exomethylene on C14 and the tertiary alcohol on C18. The relative stereochemistry of the decalin cores was assigned by comparison of the NMR data with known marine natural products. The relative stereochemistry of the cyclic peroxide moiety was proposed performing ROESY experiments and following Capon and Macleod’s empirical rules [6]. In addition, the absolute stereochemistry of the cyclic peroxide moiety was defined as 2*R*,3*S*,6*R* using the Mosher’s method [50]. It is noteworthy that two years later, the same author with two others, published the same compound from the same sponge but they named it differently, megaspinoxide A [62]. 

## 3. Marine Endoperoxide Terpenes

### 3.1. Endoperoxide Sesquiterpenes

**Figure 18:**
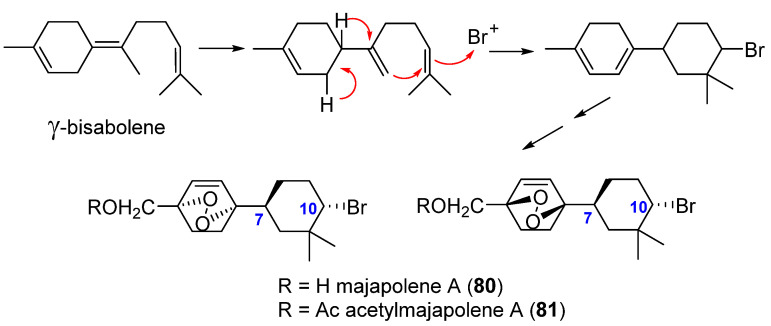
Structure and proposed biosynthesis of majapolene A (**80**) and its acetyl derivative (**81**).

In 1995, Erickson et al. [63] isolated majapolene A (**80**), a new sesquiterpene peroxide from a Philippine collection of the red algae *Laurencia majuscula* and *L. caraibica*. In both cases, majapolene A (**80**) was isolated as an inseparable diastereomeric mixture around the peroxide ring (Figure 18). The dioxabicyclo[2.2.2] structure of **80** was elucidated using different spectroscopic methods such as HREIMS, IR, and mono and bi-dimensional NMR spectroscopy. Some years later, in 2008, Vairappan et al. [64] isolated majalapone A (**80**) and its acetylated derivative (**81**), also as a mixture of diastereoisomers, from three Malaysian species of the red algal genus *Laurencia*. The absolute configuration of each diastereomer was established by vibrational circular dichroism (VCD) [65,66], indicating the absolute configuration 7*S* and 10*S* in both compounds. The authors [63] have proposed that the inseparable mixture of diastereoisomers is formed from bromonium-induced cyclization of a bisabolene system, the subsequent oxidation of the allylic methyl group and 1,4-oxygen addition of the diene system on both faces (Figure 18). Majapolene A (**80**) showed modest cytotoxicity values for all NCI 60-cell lines, a 50% net growth inhibition, relative to controls [63]. Majapolene A (**80**) and its acetyl derivative (**81**) have also displayed moderated antibacterial activities against different strains with MIC values between 10 and 40 µg/disc [64]. 

**Figure 19:**
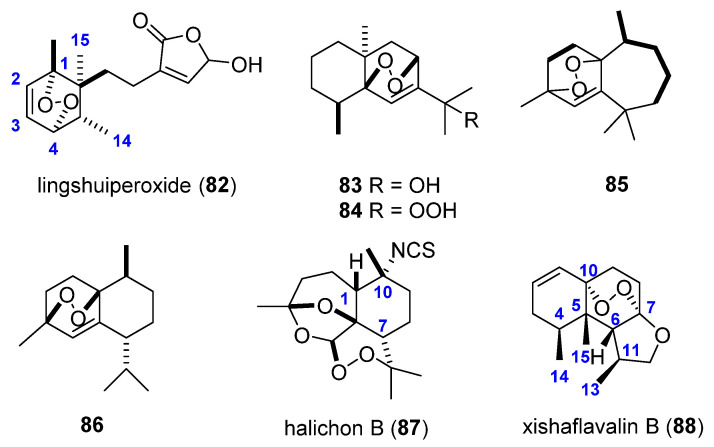
Structure and stereochemistry of different sesquiterpene peroxides.

A *Dysidea septosa* sponge collected from Lingshui Bay (China), returned a new sesquiterpene named lingshuiperoxide (**82**) (Figure 19) [67]. Its 1D and 2D NMR spectra showed one γ-hydroxyl-bearing butanolide moiety, one cyclohexene ring and a peroxide bridge linking C1 with C4. The relative stereochemistry of H14 and H15 was assigned as *cis* (both α) by comparison with lingshuiolides A and B described in the same publication. The ROESY spectrum established that the peroxide bridge has also an α-orientation and the coupling constant between H2 and H3 indicated the cis geometry of the double bond. 

In 2007, Cheng et al. [68] described the structural elucidation of two new sesquiterpenes, 5β,8β-epidioxy-11-hydroxy-6-eudesmene (**83**) and 5β,8β-epidioxy-11-hydroperoxy-6-eudesmene (**84**), from the soft coral *Nephthea erecta* collected near Green Island located on the southeast coast of Taiwan (Figure 19). The structures of these compounds were elucidated by extensive spectroscopic analyses. The relative configuration was established by NOESY experiments, and it was supported by a computer-modelled structure using MM2 force field calculations for energy minimization. Authors reported that compound **84** exhibited significant cytotoxicity against P-388 (mouse lymphocytic leukemia) and HT-29 (human colon adenocarcinoma) cells, with ED_50_ values of 0.2 and 0.4 µg/mL, respectively. Three years later, these two sesquiterpenes (**83** and **84**) were also isolated from *Nephthea* sp. collected in Lankayan Island in the state of Sabah (Malaysia) [69]. In antimicrobial assays, compound **84** showed greater antimicrobial activities against a wide range of pathogenic microorganisms than **83**. Additionally, compound **84** exhibited stronger cytotoxicity (IC_50_ = 0.36 mg/mL) against P-388 cells than compound **83** (IC_50_ = 2.2 mg/mL), perhaps because of the additional hydroperoxy moiety which plays an important role in the activity.

The diethyl ether fraction of the Saudi Red Sea soft coral, *Litophyton arboretum*, afforded a new himachalane-type sesquiterpene (3α,6α-epidioxyhimachal-1-ene, **85**) (Figure 19) [70]. Chemical structure was identified by spectroscopic measurements including 1D and 2D NMR, and the stereochemistry of the asymmetric carbons was deduced from NOESY spectrum and comparison with previous studies of β-himachalene-sesquiterpenes-stereochemistry [71]. 

In 2016, Roy et al. [72] described the structural elucidation of **86** (Figure 19), an endoperoxy cadinane-type sesquiterpenoid isolated from an Okinawan soft coral, *Sinularia* sp. Detailed analysis of the 1D and 2D NMR data confirmed the structure of **86** and 1D NOE experiments, as well as a computer-generated model using MM2 force calculations, allowed the assignment of the relative configuration of this compound. Antibacterial activity assays showed positive inhibition results of **86** against *Staphylococcus aureus* and *Salmonella enterica*. This compound also showed anti-inflammatory action in macrophage cells and cytotoxic activity against HCT 116 human colon cancer cells (IC_50_ = 43.06 µM) [72]. In addition, it has been reported that this antitumor activity by apoptosis-induction playing the H_2_O_2_ has an important role as a trigger of apoptosis [73]. The authors have proposed that these peroxy compounds are probably reduced in biological systems and therefore H_2_O_2_ is generated through a free radical reaction.

Biosynthetically related with **86**, halichon B (**87**) (Figure 19), a sesquiterpene isothiocyanate, was isolated from the Thai marine sponge *Halichondria* sp. in 2016 [74]. Compound **87** could be biogenetically derived from (−)-10-isothiocyanato-4-cadinene by oxidative cleavage of the double bond followed by different hydroxylations and cyclic hemiketal formation. Its structure was elucidated by comparison with its analog axiplyn A [75] but shows different relative configuration in the chiral centers C1, C7, and C10 as seen in NOESY experiments.

Xishaflavalin B (**88**) (Figure 19) was isolated in 2019 [76] in an elaborated chemical investigation of the Chinese soft coral *Lemnalia flava*. The nardosinane-type skeleton was determined based on extensive spectroscopic data analyses and NOESY correlations which allowed the identification of the relative configuration, locating the methyl groups C13, C14, and C15, together with H6, on the same face (β-orientation) and the peroxide group on the opposite face (α-orientation). The results obtained from the time-dependent density functional theory electronic circular dichroism (TDDFT-ECD) did not yield valid results for the determination of the absolute configuration of xishaflavalin B (**88**), due to the lack of a strong chromophore near the chiral centres. On the basis of biogenetic consideration, the absolute stereochemistry of xishaflavalin B (**88**) was tentatively assigned as 4*S*,5*S*,6*R*,7*S*,10*S*,11*S*.

### 3.2. Endoperoxide Diterpenes

**Figure 20:**
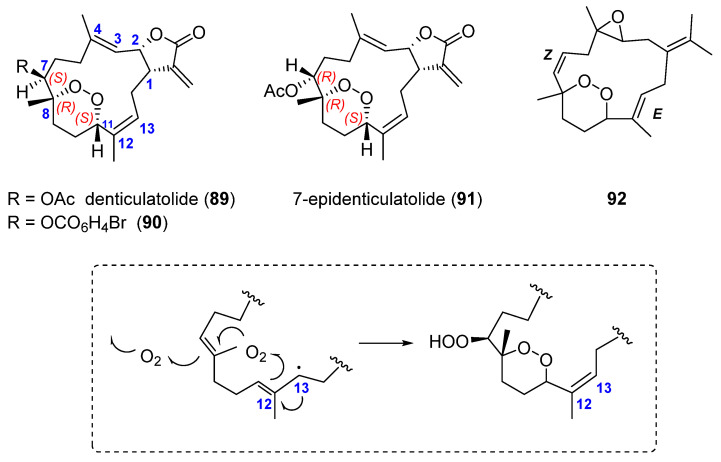
Cembranoid diterpenes and formation of cyclic peroxide.

It is well known that marine coelenterates such as soft corals (alcyonarians) are a rich source of terpene metabolites, especially cembrane-type diterpenes, which are naturally occurring compounds containing a 14-carbon ring [77]. The flexible macrocycle skeleton could reasonably be supposed to be derived from the cyclization of geranylgeranyl pyrophosphate [78]. In 1985, Uchio et al. [79] isolated the first example of a natural cembranoid having an epidioxy moiety, named as denticulatolide (**89**) (Figure 20). It was the major metabolite from the soft coral *Lobophytum denticulatum*, collected near Miyako island of Okinawa, Japan. The structure was determined by spectral and chemical evidences. Furthermore, a single-crystal X-ray analysis was carried out on a suitable derivative, confirming a *cis*-fused α-methylene-γ-lactone ring, an acetoxy group located at C7 and two trisubstituted double bonds in C3 (*E*) and C12 (*Z*). The relative configuration was also established as (7*S*,8*R*,11*S*). An *S* absolute stereochemistry at C7 was unambiguously established by X-ray crystallography performed with the *p*-bromobenzoate **90** [80]. The complete ^1^H-^13^C assignment of this cembranolide was fulfilled by 2D-experiments and published some years later [81]. Fukazawa et al. [82] proposed that in the most stable conformation of **89**, the γ-lactone ring and the 14-membered carbocycle adopt an almost planar arrangement. The biosynthesis of **89** is unknown, but the presence of the uncommon 12-*cis* double bond suggests a pathway similar to that found in the biosynthesis of prostaglandins [83]. Namely, formation of a radical at C13 of the ordinary cembrane skeleton followed by the addition of two oxygen molecules would lead to the endo-peroxide structure (Figure 20) [84].

Denticulatolide (**89**) has attracted attention due to its wide range of biological activities, such as its analogues cembranes. It is produced as a defence against predators, showing ichthyotoxic properties [79]. It also possess significant cytotoxic activity toward P-388 cell line (mouse lymphocytic leukemia) [81], HL-60 cell line (human promyelocytic leukemia) [85], and other cell lines [80,86,87]. In addition, the α-methylene-γ-lactone moiety present in its structure plays an important role in the inhibitory effect on nitric oxide production, contributing to the development of therapies for anti-inflammatory diseases [85,86,87,88]. This interesting compound has also been reported to be present in different species of soft corals such as *Sinularia mayi* [84], *Sarcophytum crassocaule* [81], *Sinularia gibberosa* [89], and *Lobophytum crassum* [86]. 

The search for other biologically active compounds of soft corals has led to the isolation of a highly oxidized cembranolide: 7-epidenticulatolide (**91**) (Figure 20), which was obtained as a minor metabolite of *Lobophytum denticulatum* [90]. The structure was deduced spectroscopically, and compound **91** is a stereoisomer of **89**, showing only differences regarding the C7 configuration. The X-ray analysis defined the structure of **91** and gave the relative stereochemistry of the molecule. Another cembranoid diterpene **92** (Figure 20) was obtained from a new species of *Lobophytum* soft coral collected from the South Andaman Coast in the Bay of Bengal, India. The structure was determined from spectral data and by comparison with compound **89** [91]. 

**Figure 21:**
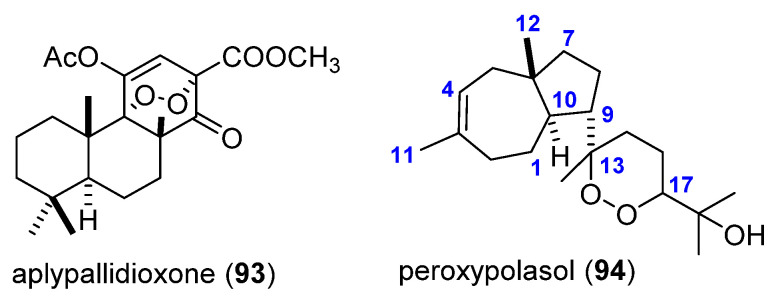
Endoperoxides aplypallidioxone and peroxypolasol.

Aplypallidioxone (**93**) (Figure 21) was isolated from a new encrusting sponge, conveniently labelled *Aplysilla pallida* [92]. Its structure was elucidated by extensive spectroscopic (MS, UV, ^1^H NMR, ^13^C NMR and 2D NMR) data analysis and by comparison with a similar compound, aplypallidenone, that was also isolated from the same sponge and its crystal structure was determined by X-ray diffraction method. The functionalization of ring C in compound **93** is reminiscent of compounds already reported from *Spongia officinalis* [93]. A possible pathway to compound (**93**) starts from isoagatholactone that was the first reported member of the diterpene spongian family. 

Diterpene peroxypolasol (**94**) [94] (Figure 21) has been isolated from the Japanese marine sponge *Epipolasis* sp. (Jaspidae). Its structure was determined on the basis of spectroscopic data. These data require **94** to contain a seven/five-membered ring and a peroxide ring in the side chain. The relative stereochemistry was established by NOESY experiments. The NOE between H12/H1β, H4, H5β, H7β, H9β; and between H5α/H2, H10 established the trans A/B ring junction, the β-orientation of H9, and the α-orientation of H-10. The configuration at C17 was undetermined because of an ambiguous configuration at C13 (configurations at C13/C17; *S*/*R* or *R*/*S*).

## 4. Biological Activity Overview

So far, more than 90 marine terpenic endoperoxides have been described, the vast majority being norsesterterpenes (59 compounds) and norditerpenes (17 compounds). Other terpenoids with an endoperoxidic moiety have also been isolated, although these are less frequent, like those having a sesqui- or diterpene structure (14 compounds).

There are many reports on the biological activity, possibly stimulated by the attractive endoperoxide moiety present in these substances. The studies have mainly focused on their activity against microorganisms, such as bacteria, yeasts, or viruses, as well as against protozoa that cause diseases, especially *Plasmodium*, which causes malaria, or *Trypanosoma* (Table 1). In addition, measurements of in vitro activity against different tumour cell lines are well documented (see Table 1 for references). To a lesser extent, the anti-inflammatory activity has also been measured.

Both norsterterpenes and norditerpenes exhibit a remarkable range of bioactivities, although the number of studies with the first type is superior, possibly due to the fact that norsesterterpenes are more abundant. In all cases, the bioactive compounds are the free carboxylic acids, although sometimes natural methyl esters have been isolated. For example, although the acids **57** and **58** show activity against the bacteria and yeasts tested, their corresponding methyl esters, **57a** and **58a**, are inactive [46].

It is interesting to notice that the presence of the free acid form of these metabolites and the configuration of the methyl group in C6, may be critical for their growth of inhibitory activity in various cancer cell lines in vitro [12,31]. In the case of the NO inhibition related to inflammatory processes, both the presence of a free carboxylic acid and an equatorial orientation of the methyl group attached to C6 are critical for increased activity [33].

Of all the isolated compounds, the most tested and the one with the greatest range of activities is (+)-muqubilin (**12**) and its enantiomer **21**. This compound is active against *Plasmodium*, and this is the reason why it has been used as a prototype for the development of new antimalarial agents [23,24]. In addition, it is effective against *Troxoplasma gondii*. Moderate activity against the *Nicotiana tabacum* plant has also been described, as a result of a program devoted to the search of natural marine products as prototypes for agrochemical agents [26].

Finally, it is also worth mentioning that diterpene **89** and the acid fraction of *Sigmosceptrella laevis*, from which **50**, **51**, and **52** were isolated, also have ichthyotoxic properties as a defence method against predators [79].

## 5. Conclusions

Marine macro- and microorganisms, especially algae, soft corals, sponges and fungi, have a metabolism that produces a great diversity of bioactive compounds, many of them unparalleled in the terrestrial environment. The composition of many species of sponges from tropical and temperate waters has been investigated, with a wide diversity of compounds found in their extracts. Among them, a group of substances stands out: those with a norterpene skeleton, that is, terpenoids that have one carbon atom less than what would be produced through an ordinary biosynthesis, and that additionally incorporate an endoperoxide function. These substances not only have structures of moderate complexity, but they also present numerous asymmetric centres. The structural identification of these compounds is very complex, especially from the point of view of the stereochemical assignment of the chiral centres, that has required the development of remarkable special techniques. The group of substances presented in this review, originating from marine invertebrates, such as sponges, is exceptional due to the range of potential pharmacological applications. The antitumor activity of some of these substances stands out, some of which are capable of inhibiting the tumour cell growth in in vitro cultures at concentrations between 10 and 100 µg/mL. It is certainly a very promising field of research that will continue to provide exceptional developments in the near future.

## Figures and Tables

**Table 1:** Biological activities of marine terpene endoperoxides.

Target	Norsesterterpenes			Norditerpenes ^d^	Sesqui- and Diterpenes ^e^
	Acyclics ^a^	Monocyclics ^b^	Bicyclics ^c^		
Bacteria	**1** [6], **3a** [9], **4** [9]	**12** [20]	**57** [46], **58** [46], **61** [47], **67** [51], **68** [51], **79** [61]		**80** [64], **81** [64], **84** [69], **86** [72]
Yeasts	**1** [6]	**12** [20]	**57** [46], **58** [46], **61** [47], **79** [61]		
Viruses	**5** [11]	**12** [11]	**67** [51], **68** [51]		
*Plasmodia*		**12** [24], **15** [24,28], **20** [24,28], **21** [39], **45** [39], **45a** [39], **46** [39], **47** [39]	**50** [23], **55** [28]	**8** [24,28], **9** [15], **13** [39], **19** [24,28], **30** [39], **31** [15], **32** [15]	
*Trypanosoma*	**10** [16], **11** [16]	**22** [16]	**50** [16], **50a** [16], **51** [16]	**26** [16]	
*Toxoplasma*		**12** [11]	**51** [11]		
Inflammation		**22** [33,34]			**86** [72], **89** [84,85,86,87]
Citotoxicity	**5** [10,12]	**15** [27], **16** [27], **17** [27], **21** [31], **38** [36]	**50** [33], **51** [12], **51a** [12], **67** [51], **68** [51], **74** [58], **75** [14], **76** [14], **77** [14], **78** [14], **79** [61]	**6** [10], **7** [10], **13** [22], **18** [12], **25** [13]	**80** [63], **83** [69], **84** [68,69], **86** [72,73], **89** [79,80,84,85,86]

^a^ 6 described structures, 6 bioactive. ^b^ 22 described structures, 11 bioactive. ^c^ 31 described structures, 14 bioactive. ^d^ 17 described structures, 12 bioactive. ^e^ 14 described structures, 6 bioactive.
